# Molecular Cloning and Characteristics of a Lectin from the Bivalve *Glycymeris yessoensis*

**DOI:** 10.3390/md21020055

**Published:** 2023-01-17

**Authors:** Tatyana O. Mizgina, Sergey N. Baldaev, Galina N. Likhatskaya, Valentina I. Molchanova, Maxim S. Kokoulin, Alina P. Filshtein, Eugene A. Rogozhin, Irina V. Chikalovets, Marina P. Isaeva, Oleg V. Chernikov

**Affiliations:** 1 G.B. Elyakov Pacific Institute of Bioorganic Chemistry, Far Eastern Branch of Russian Academy of Sciences, Vladivostok 690022, Russia; 2 Shemyakin-Ovchinnikov Institute of Bioorganic Chemistry, Russian Academy of Sciences, Moscow 117997, Russia

**Keywords:** bivalve lectins, hemolymph, C-type lectins, glycosylation, multigenic family

## Abstract

C-type lectins (CTLs) are a family of carbohydrate-binding proteins that mediate multiple biological events, including adhesion between cells, the turnover of serum glycoproteins, and innate immune system reactions to prospective invaders. Here, we describe the cDNA cloning of lectin from the bivalve *Glycymeris yessoensis* (GYL), which encodes 161 amino acids and the C-type carbohydrate recognition domain (CRD) with EPN and WND motifs. The deduced amino acid sequence showed similarity to other CTLs. GYL is a glycoprotein containing two N-glycosylation sites per subunit. N-glycans are made up of xylose, mannose, D-glucosamine, 3-O-methylated galactose, D-quinovoses, and 3-O-methylated 6-deoxy-D-glucose. The potential CRD tertiary structure of the GYL adopted CTL-typical long-form double-loop structure and included three disulfide bridges at the bases of the loops. Additionally, when confirming the GYL sequence, eight isoforms of this lectin were identified. This fact indicates the presence of a multigene family of GYL-like C-type lectins in the bivalve *G. yessoensis*. Using the glycan microarray approach, natural carbohydrate ligands were established, and the glycotope for GYL was reconstructed as “Galβ1–4GlcNAcβ obligatory containing an additional fragment”, like a sulfate group or a methyl group of fucose or N-acetylgalactosamine residues.

## 1. Introduction

Bivalves are a group of marine fauna vulnerable to various pollutants and aquatic microorganisms as they are sedentary filtering organisms. Due to the lack of antibody-based immunity in invertebrates, the protection of mollusks from pathogenic infection depends solely on various pathogen recognition receptors (PRRs). An analysis of marine bivalve mollusk genomes revealed a very complex immune system in which PRR homologs, the basis of vertebrate innate immunity, are encoded by large multigenic families [1,2]. Hemolymph, the circulatory fluid of invertebrates, contains hemocytes, which secrete effector molecules with immune regulatory functions. PRRs such as lectins, cytokines, nitric oxide synthases, and antimicrobial peptides compose the innate immune system of mollusks [3,4]. Among them, lectins play a decisive role due to their ability for highly specific recognition of various carbohydrates located on the cell surface of microorganisms—pathogen-associated molecular patterns (PAMPs), a group of molecules characteristic of pathogens but absent in the host organism. The recognition of patterns of pathogenicity and the stimulation of a series of protective immune responses are the basis of innate immunity [5]. Due to the diverse carbohydrate specificity of lectins present in the body of a single animal, it becomes possible to identify a wide range of pathogens. Lectins belong to a heterogeneous group of oligomeric proteins differing in several aspects, such as structure, size, molecular organization, and, especially, carbohydrate specificity. These properties determine the presence of the various types of biological activity of lectins, including antitumor [6], antifungal [7], antibacterial [8], and antiviral [9,10,11] activities.

The classification of all existing lectins is based on the similarity in the amino acid sequence and the structural arrangement of the carbohydrate recognition domain (CRD), as well as the ligan-binding specificity [12]. The main families are C-type lectins, F-type lectins, R-type lectins, M-type lectins, P-type lectins, X-type lectins, I-type lectins, pentraxins, galectins (formerly S-type lectins), calnexins, and tachylectins [13]. The affinity and specificity of lectins depend on a unique set of amino acids included in the CRD and interacting with carbohydrate ligands through hydrogen bonds and stacking interactions. The avidity of the lectin–ligand interaction can increase as a result of the multivalent binding of CRDs of lectin subunits with multivalent ligands [14].

The best-known C-type lectins (CTLs) share a common CTL-like domain (CTLD). The binding to a variety of carbohydrate ligands occurs in the presence of calcium ions in most CTLD-containing proteins. The domain includes the key residues involved in sugar binding located on a long flexible loop and four critical cysteine residues, which stabilize a two-loop structure [15]. The key conserved residues that bind sugars contain EPN (Glu-Pro-Asn) first motif (which promotes binding to Man, GlcNAc, Fuc, and Glc) or QPD (Gln-Pro-Asp) motif (which promotes binding to Gal and GalNAc). The second motif is WND (Trp-Asn-Asp), which has been identified to cooperate with the first motif in the binding process against carbohydrates. However, in invertebrates, these two motifs are highly diverse, and different amino acid variants occur at the corresponding sites of these motifs. For example, QPG (Gln-Pro-Gly), EPD (Glu-Pro-Asp), YPG (Tyr-Pro-Gly), QPS (Gln-Pro-Ser), and YPT (Tyr-Pro-Thr) were found in the first motif. At the same time, the following sequences were observed in the second one: WSD (Trp-Ser-Asp), WID (Trp-Ile-Asp), FSD (Phe-Ser-Asp), WHD (Trp-His-Asp), and LSD (Leu-Ser-Asp). Motives play an important role in the final stability of the protein interacting with carbohydrates and Ca^2+^ ions. The variety of motifs shows that lectins with different affinities are capable of a wide spectrum of recognition [16,17].

In recent years, many CTL genes have been identified and cloned from invertebrates due to the significance of C-type lectins in the innate immunity [18]. The accumulated research on invertebrate C-type lectins has focused especially on insects, crustaceans, and mollusks [19,20]. In bivalves, several C-type lectins were cloned or purified from clams [21], oysters [22,23,24], and scallops [25,26].

Previously, we identified probably a new member of the C-type lectins family from the hemolymph of the bivalve *Glycymeris yessoensis* (GYL). GYL is a dimeric protein with a molecular mass of 36,053 Da, consisting of 18,118 Da subunits linked by a disulfide bridge. It recognizes glycoproteins containing O-glycosidically linked glycans and may be involved in the immune response of *G. yessoensis* to bacterial attack [27]. In this paper, we report a full-length sequence of GYL obtained by 3′- and 5′-RACE strategy. Data on the specificity and affinity of GYL for various oligosaccharides are obtained using a glycan microarray. Moreover, we reveal several different GYL-like transcripts, which are probably encoded by a multigene family.

## 2. Results and Discussion

### 2.1. Determination of a Full-Length Sequence of GYL and Analysis of Its Isoforms in the Bivalve G. yessoensis

**Figure 1:**
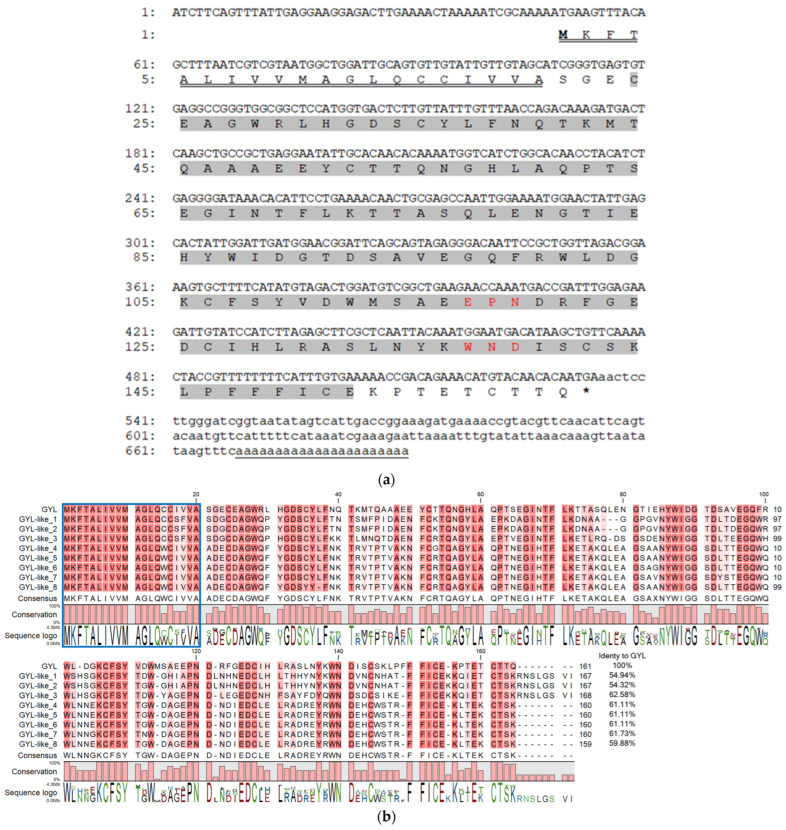
(**a**) cDNA sequence and deduced amino acid sequence of GYL. Nucleotides and amino acids are numbered to the left of the sequences. Start codon is ATG; stop codon is TGA. The 5’- and 3’-UTRs are shown in lower-case letters. The 690 bp cDNA sequence comprises a 48 bp, 5′ untranslated region UTR, a 156 bp 3′ UTR includes the poly(A) tail, and a 483 bp ORF that encodes a protein of 161 amino acids. The single CTLD predicted by the SMART program highlighted in grey was located at position 24–152 and included conserved EPN (Glu117-Pro118-Asn119) and WND (Trp137-Asn138-Asp139) motifs (highlighted in red). The representative amino acid sequences (CFSYVDWMSAEEPNDRF, LPFFFLCEKPTETC, and WNDLSCSK) identified with nano-ESI MS/MS were also found in the amino acid sequence encoded by the ORF [27]. The poly(A) signal is underlined with one line. A letter underlined with double line indicates a predicted signal peptide. (**b**) Full-length GYL-like proteins from *G. yessoensis*. Identical and conserved amino acid residues are shown on a dark- and light-red background, respectively. Vector NTI Advance ™ 11.0 (Invitrogen, Carlsbad, CA, USA) [30] was used for multiple sequence alignment. Visualization via software CLC Main Workbench (Qiagen, Aarhus, Denmark).

To determine the primary structure of GYL, we cloned a full-length transcript using 3′- and 5′-RACE with degenerate primers developed on their peptide sequences [27]. Firstly, step-out 3′-RACE with the two forward Lectin_PTSE and Lectin_MMD primers resulted in ~450 bp cDNA encoding the EPN- and WND- motifs, stop codon, and a poly(A) tract. This sequence was further used to design the gene-specific primers, GYL_R1_Cap and GYL_R2_T7, for step-out 5′-RACE. As a result, a fragment of ~660 bp coding a 5′-untranslated region, a signal peptide, and an N-terminal fragment of a mature protein was obtained (Figure 1a). The resulting GYL sequence of 690 bp was assembled from the overlapping RACE fragments.

To confirm this sequence, GYL_start and GYL_stop1 primers were designed from the flanking sequences of GYL. After cloning and sequencing the ~700 bp PCR fragment, different *GYL-isoforms* were revealed—*GYL-isoform1–GYL-isoform9* (Figure 1b). The length of the cDNAs varied from 690 bp (*GYL-isoform8*) to 717 bp (*GYL-isoform3*). The deduced amino acid sequences of the *GYL-isoform1–GYL-isoform9* were compared. We denoted GYL-isoform1 as GYL due to it containing/sharing the same peptide sequences as those identified with nano-ESI MS/MS [27]. The other GYL isoforms were named GYL-like 1–8. All isoforms have a high conservative signal peptide at position 1–21 aa, and a high variable sequence of mature proteins (54.32–61.73% of sequence identity, Figure 1b). The molecular weight of mature proteins ranged from 15,996 to 16,862 Da, and the pI values ranged from 4.39 (GYL-like 3) to 5.15 (GYL-like 1) (Table 1). A common feature of GYLs is that all mature proteins contain a single CTLD at position 24–152 aa, predicted by the SMART program, and include conserved EPN or APN (GYL-like 1 and GYL-like 2) and WND motifs (Figure 1b). The lectins can be attributed to CTLs.

The presence of a conserved signal peptide and a variable mature protein sequence is characteristic of sequences encoded by multigenic families [28,29]. This fact directly indicates the existence of a multigene family of GYL-like C-type lectins in the bivalve *G. yessoensis*.

The family of C-type lectins is characterized by low sequence similarities despite a conserved secondary structure and a well-conserved fold. We used the SPOMA online program to predict the secondary structures of GYL and eight GYL-like proteins (Table 1). We found that the coils were a major component of the secondary structure of GYL-like proteins, and the content was 53.96–60.54%. The secondary structure α-helix of GYL-like proteins accounted for a proportion of 15–17%. The proportion of extended chains in GYL-like proteins structure was 23–28%.

Previous circular dichroism studies have established that a characteristic feature of GYL is the predominance of the β-structure [27]. The prediction of the secondary structure of the protein based on its amino acid sequence revealed an increase in α-helices content. Such differences may most likely be due to the glycosylation of lectin, which plays a significant role in protein folding and enables stabilization. The effect of glycosylation on protein depends on the location, chemical environment, and the number of glycosylated sites in the protein [31]. The use of the prediction algorithm for the spatial structure of a protein makes it possible to obtain more reliable results because, in this case, the influence of environmental factors such as solvent, temperature, etc., is excluded.

In addition, a comparative analysis of nine members of the GYL-like proteins’ secondary structure indicates high structural similarity with a range of vertebrate CTLDs (more than 30%), such as the ones from scavenger receptor [32], mincle protein [33], CD209 antigen-like protein B [34], and of invertebrate CTDL Codakine [35]. These proteins are the PRRs and are critical for innate immunity, similar to human DC-SIGN/CD209, binding a broad variety of microbial organisms and their polysaccharides.

### 2.2. Characteristics of GYL

#### 2.2.1. Homologous Analysis of GYL

The BLAST analysis revealed significant sequence similarity between CTLD of GYL and CTLD-containing proteins and lectins. For example, GYL shared 42% similarity with an unnamed CTLD-containing protein from the gastropod *Candidula unifasciata* (CAG5124220.1), 41% with an unnamed CTLD-containing protein from the bivalve *Mytilus coruscus* (CAC5386982.1), 40.8% with macrophage mannose receptor 1 from the snow crab *Chionoecetes opilio* (KAG0712468.1), 39.5% with a perlucin-like protein (XP_034305914.1) from *Crassostrea gigas*, and 38.17% with a CTLD-containing protein (CLECT_DC-SIGN_like) from the Tasmanian devil *Sarcophilus harrisii* (XP_012396311.1).

**Figure 2:**
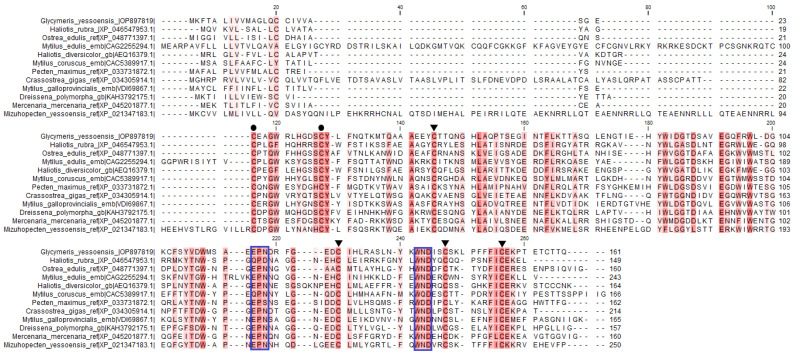
Multiple sequence alignment by ClustalW of GYL with other CTLD-containing proteins and lectins. GenBank accession numbers for some of the species included are as follows: *M. galloprovincialis* (VDI69867.1), *Ostrea edulis* (XP 048771397.1), *C. gigas* (XP 034305914.1), *Haliotis rubra* (XP_ 046546494.1), *Haliotis diversicolor* (AEQ16379.1), *Pecten maximus* (XP_033731872.1), *Mercenaria mercenaria* (XP_045201877.1), *Dreissena polymorpha* (KAH3792175.1), *M. coruscus* (CAC5389917.1), *M. edulis* (CAG2255294.1), and *Mizuhopecten yessoensis* (XP 021347183.1). Amino acid residues shaded in dark red are conserved for at least 50% sequences, and similar amino acids are shaded in light red. Cysteine residues marked with ▼ are involved in the formation of the CRD internal disulfide bridges, whereas two extra cysteine residues in the long-form are marked with ●. The motif for determining ligand-binding specificity is indicated with a frame. Visualization via software CLC Main Workbench (Qiagen, Aarhus, Denmark).

The signature sequences of the C-type lectin superfamily were identified in GYL by multiple sequence alignment. Disulfide bonds of mature proteins play a critical role in the folding and refolding of proteins and in establishing and maintaining three-dimensional structures. The four cysteine residues (C1, C2, C3 and C4) involved in the formation of the internal disulfide bridges were well conserved in the CRD (Figure 2). In addition, another two cysteine residues (C0 and C0’) were located at the N-terminus of each CRD, indicating that the CRD in GYL was of long-form (Figure 2).

#### 2.2.2. Signal Peptide of the GYL Sequence

Signal peptide (SP) prediction based on GYL sequence data was performed using the SignalP 6.0 SP detection program [36]. Signal peptide GYL contains two characteristic sequences for the cleavage site (Val18-Val19-Ala20 и Ala20-Ser21-Gly22). The cleavage site between the amino acids Gly22 and Glu23 was predicted with a probability of 94%, and the cleavage site between Ala20 and Ser21 was predicted with a probability of 6%. Using this program, the type and subregions of SP were determined, which characterize each amino acid of SP. The consensus sequence for the cleavage of the signal peptide from a secreted protein was established as Ala-X-Ala (-3, -2, or -1), -1 can accommodate Ala, Ser, Gly, Cys, Thr, or Gln, while the -3 residue cannot be aromatic (Phe, Tyr, or Trp), charged (Asp, His, Glu, Lys, or Arg), or large polar (Asn or Gln) amino acids. The position -3 can also contain larger residues such as Val, Thr, Leu, and Ile. Alanine is the most common amino acid residue at positions P3 (>50%) and P1 (>80%) [37]. To determine the exact site of proteolysis, Edman sequencing of the N-terminal fragment of GYL was performed. The 18,118 Da protein was transferred onto a PVDF membrane and subjected to N-terminal protein sequencing by Edman degradation. As a result, the N-terminal amino acid sequences Ser-Gly-Glu-X-Glu-Ala-Gly-Trp and Glu-X-Glu-Ala-Gly-Trp (X indicates an unidentified amino acid residue) were obtained. It is possible that the studied sample is a mixture of two GYL isoforms that differ by two N-terminal amino acid residues. Alternatively, the heterogeneity of the N-terminus may have arisen because of the uneven cleavage of the SP, in view of the presence of two characteristic sequences for cleavage (Val18-Val19-Ala20 and Ala20-Ser21-Gly22).

A short N-terminal peptide, called a signal peptide, is present in the vast majority of secretory proteins. SPs control the rate of protein secretion by determining the state of protein folding, influencing downstream transmembrane behavior and N-terminal glycosylation, nuclear localization signals, and playing a role in the infectious potential of bacteria and viruses [38]. The presence of SP in the GYL amino acid sequence indicates that it is a secreted molecule. Probably, lectin is secreted by hemocytes into the mollusk hemolymph and functions as an innate immunity component.

#### 2.2.3. N-Glycosylation of GYL

It is known that the most common post-translational modification is protein glycosylation, which not only affects stability, structure, and folding but also controls the main biological pathways, ranging from protein transfer and cell adhesion to the host–pathogen interaction [39].

Comparing the molecular weight of the amino acid sequence obtained from cDNA with the mass spectrometry data, we noted that it is 1089 Da less. Because different lectins are glycosylated, we decided to analyze whether there are sugars in the GYL structure. Gas–liquid chromatography of GYL N-glycans in the form of acetylated methyl glycosides showed the presence of monosaccharide residues of xylose, mannose, D-glucosamine, 3-O-methylated galactose, 6-deoxy-D-glucose (D-quinovoses), and 3-O-methylated 6-deoxy-D-glucose. To date, xylose and methylated hexoses have been found in the N-glycans of proteins in many mollusk species and are recognized as their typical feature [40]. Using the NetNGlyc-1.0 server to predict N-glycosylation sites in proteins, it was determined that, in the amino acid sequence of GYL, Asn19 and Asn60 are N-glycosylated. Thus, there are two glycosylation sites per GYL subunit and four glycosylation sites per dimeric molecule.

It is known that asparagine-linked N-glycosylation is the most recurrent form observed in eukaryotes. This occurs almost always when the asparagine residue is followed by the XT/S sequon, where X refers to any amino acid residue except proline [41]. Investigations of invertebrate glycosylation are limited, fragmentary, and unsystematic, while glycosylation in mammals is being extensively studied. In recent years, a wide variety of invertebrate species and an increase in the database of sequenced genomes proteins has led to an increase in the number of studies that have focused on glycomes derived from invertebrate species. It has been shown that in snails, the most common N-linked glycans are oligomannosidic and small paucimannosidic structures, sometimes ending in 3-O-methylated mannoses. These studies may help to identify the carbohydrate epitopes, which may be relevant for immune responses [42]. It was determined that lectin from the mussel *M. californianus* is a glycoprotein with high α-mannose glycans and sugars terminated in sialic acid linked with α(2–6) or α(2–3) to galactose [43].

The bacterial-infection-resistant mutant *Caenorhabditis* is deficient in many N- and O-glycans compared to its wild type. In invertebrates, the disruption of glycosylation pathways has been shown to cause serious defects such as abnormal metamorphosis and even mortality. The study of glycosylation in invertebrates often provides new insights into the mechanisms underlying physical/neurological disorders in vertebrates and helps to develop new therapeutic treatment strategies [41].

#### 2.2.4. The Potential Tertiary Structure of GYL

**Figure 3:**
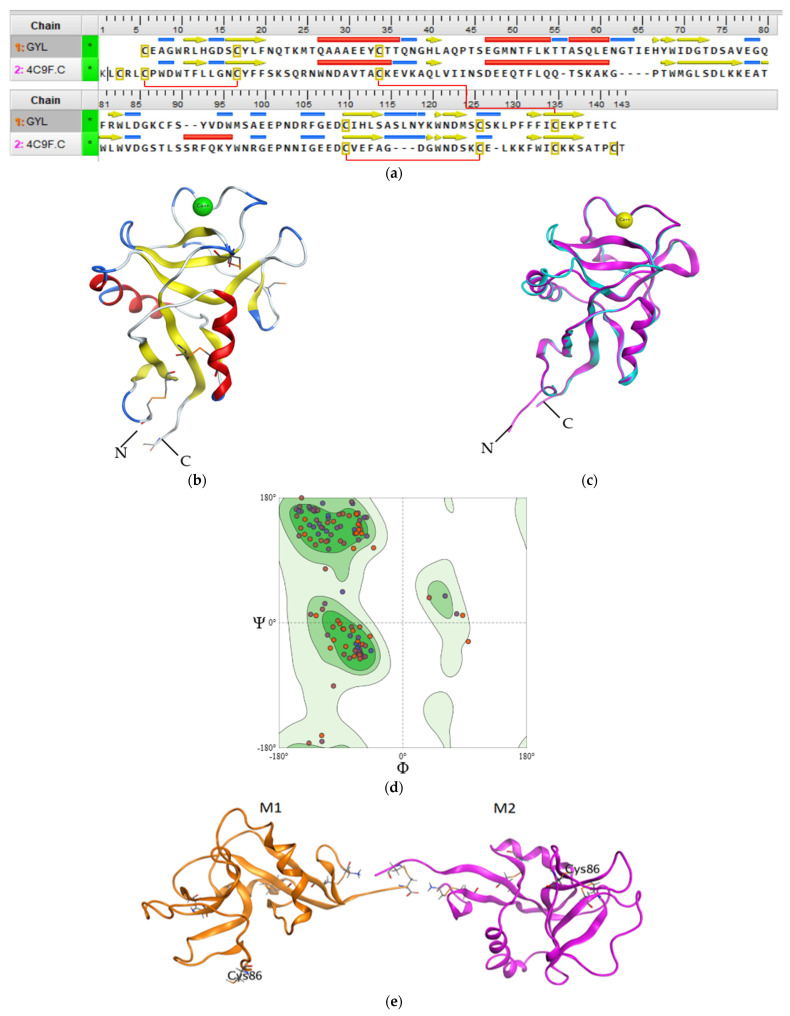
(**a**) Sequence alignment of GYL and mouse CD209 (PDB ID 4C9F). Intramolecular disulfide bridges are shown with red lines. (**b**) The 3D structure homology model of GYL predicted by SWISS-MODEL server with disulfide bonds displayed as orange sticks. There are four cysteines C1–C4 and C2-C3 involved in forming disulfide bridges at the bases of the loops. The cystine bridge specific for long-form CTLDs C0-C0′ is also shown. (**c**) 3D superimposition of the GYL homology model (blue) and template mouse CD209 (pink). (**d**) Ramachandran plot for the validation of the lectin model. (**e**) The putative model of the GYL dimer 3D structure. M1 and M2 are subunits of GYL. Cys132 is the putative residue involved in the unique dimerization mode observed in this C-type lectin. The figure was received using the program MOE 2020.0901.

Regardless of the low GYL sequence similarity (20–30%) with other C-type CRDs and CTLDs, a structural comparison with known three-dimensional structures by the SWISS-MODEL server revealed that GYL is very similar to long-form CTLD, such as a mouse CD209 antigen-like protein (PDB ID 4c9f.1) [34]. The potential tertiary structure of CRD in GYL was established by the SWISS-MODEL prediction algorithm based on the template 4c9f.1 (mouse CD209 antigen-like protein). Sequence identity was 30% (Figure 3a), and a root-mean-square deviation (rmsd) of 0.404 Å over 126 aligned C-alpha atoms was observed (Figure 3c).

We used QMEANDisCo global score to determine the quality of the constructed theoretical protein model. The QMEANDisCo score shows how the constructed model is comparable with the experimental structures of proteins of the same size [44]. A QMEANDisCo global score close to 1.0 indicates good agreement. The QMEANDisCo score of GYL is 0.61 ± 0.07. The stereochemical quality and reliability of the structure were tested using the Ramachandran plot and Z-score. Most amino acid residues (91.73%) were in the favorable region on the Ramachandran plot (Figure 3d). The Z-score of the protein was calculated to be −1.69 ± 0.70. The MolProbity score was 2.14.

The CRD adopted a typical long-form double-loop structure. A lower part domain was composed of two α-helices and four β-strands, and an upper part was composed of five β-strands. The Ca^2+^-binding site 2 involved in carbohydrate binding was located in the long loop region of the upper part. Six cysteines formed three disulfide bridges at the bases of the loops. Four cysteines C1(Cys32)-C4(Cys131) and C2(Cys106)-C3(Cys122), which are the most conserved CTLD residues, form two disulfide bridges at the bases of the loops. Cys32 and Cys131 linked the whole domain loop, while Cys106 and Cys122 linked the long loop region (Figure 3b). In addition, the other two cysteines C0(Cys4)-C0′(Cys15) formed the third disulfide bridge, stabilizing the lower part of the CRD, which is specific for long-form CTLDs. Twain free cysteines—Cys86 and Cys132—are putative cysteines for the formation of an intermolecular bridge. Cys86 is observed on the long loop region. Cys132 is located on the C-region protein.

The long loop region is involved in Ca^2+^-dependent carbohydrate binding, and in the domain-swapping dimerization of some CTLDs, which occurs via a unique mechanism [15].

Previously, it was shown that GYL is a dimeric protein consisting of subunits linked by a disulfide bridge. The predicted GYL tertiary structure adopted a dimeric arrangement allowing for the opposite face presentation of carbohydrate-binding sites (Figure 3e). Among the available C-type lectin crystal structures a homodimeric structure composed of two C-type single-CRDs was obtained for the tunicate (*Polyandrocarpa misakiensis*) lectin TC14 complexed with galactose [45], the sea cucumber (*Cucumaria echinata*) CEL-I lectin complexed with GalNAc [46], and the sea bivalve (*Codakia orbicularis*) Codakine lectin complexed with nonasaccharide-Asn 1 [35]. In all cases, this arrangement is more suitable for cell aggregation than for the avidity of binding.

#### 2.2.5. Affinity and Specificity for Oligosaccharides

Glycan microarray allows a high-throughput investigation of lectin specificity and glycan-binding activity [47].

**Figure 4:**
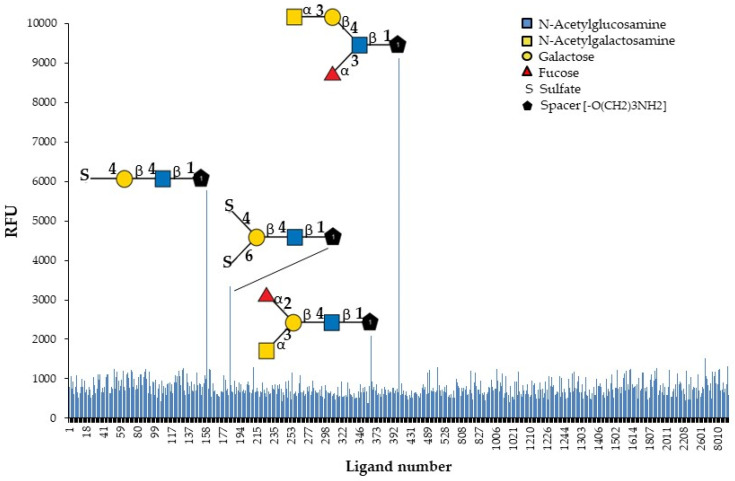
Identification of glycan ligands for GYL by glycan array data.

The identification of natural carbohydrate ligands for GYL is an important task for the subsequent study of its biological activity. GYL was tested to determine its binding profile to chip-presented substances, mostly glycans, and fragments of glycoprotein and glycolipid carbohydrate chains. The specificity of the biotin-labeled lectin was evaluated by binding to the 609 compounds present on the glycan array (Figure 4).

Only four oligosaccharides are recognized with high affinity. The results (Figure 4 and Table 2) revealed the strongest GYL binding to the GalNAcα1-3Galβ1-4(Fucα1-3)GlcNAcβ-sp3 tetrasaccharide. Additionally, GYL had a strong binding response to the 4-O-Su-Galβ1-4GlcNAcβ-sp3 epitope and a weaker response to 4,6-O-Su2-Galβ1-4GlcNAcβ-sp2 and GalNAcα1-3(Fucα1-2)Galβ1-4GlcNAcβ-sp3. However, all glycans contain the same common motif—Galβ1-4GlcNAcβ—highlighted in bold in the table. The substituents of galactose significantly increase the strength of lectin binding to the glycan. Thus, the addition of a sulfate group at position 4 to a disaccharide results in a six-fold increase in binding. However, additional sulfating at position 6 markedly reduces binding. The monosaccharide GalNAc attached to the trisaccharide Le^x^ leads to a 12-fold increase in binding. At the same time, the addition of this monosaccharide to an epitope Galβ1-4GlcNAcβ not containing Fuc leads to a great loss in activity. It should be noted that the position of the fucose group is also important. So, in the most active tetrasaccharide, the fucosylation of the GlcNAc terminal fragment (Lewis x) is acceptable but not the fucosylation of Gal. If the branching point is Gal, the binding force drops by almost four times.

Notably, neither lactosamine disaccharide as is, without substituents, nor the most common naturally occurring branched oligolactosamine, containing trimannosyl core, are positive in the glycan array. Taking into account all the mentioned features, one can reconstruct the glycotope for the GY-like “Galβ1-4GlcNAcβ obligatory containing an additional lectin contacting fragment”, apparently like a sulfate group or a methyl group of fucose or N-acetylgalactosamine residues. Lectins do not exhibit strict specificity toward one type of saccharide. This could be related to the versatility and high environmental adaptability of GYL.

GYL is unable to recognize mannose and mannose-capped glycans [27], although its CRD presents the typical structural elements of a C-type lectin-like domain, including the EPN motif commonly associated with Man/Fuc/Glc and GlcNAc selectivity like other CTLs [35,48]. Interestingly, CEL-IV, a C-type lectin isolated from the sea cucumber Cucumaria echinata, shows galactose specificity, although it contains an EPN motif in the carbohydrate-binding site. This discrepancy was explained by the crystal structure of CEL-IV complexed with galactose-containing carbohydrates, in which the aromatic residue Trp79 specifies the orientation of the bound galactose by stacking interaction so that EPN can form proper hydrogen bonds with galactose [49]. The specificities of the C-type CRDs are not only exclusively specified by the EPN or QPD motifs but also largely supported by other residues situated nearby in the binding site, and according to the data obtained, strong evidence for this has been demonstrated by the site-directed mutagenesis on the mannose-binding lectin MBP-A [50] and Gal/GalNAc-specific CEL-I [51].

The biological role and the natural ligands have not yet been defined for GYL. Previously, it was shown that GYL recognizes glycoproteins containing O-glycosidically linked glycans, such as porcine stomach mucin (PSM), fetuin, thyroglobulin, and ovalbumin, and exhibits antibacterial activity, likely binding to polysaccharides present on the surface of bacteria [27].

The growth of the mucin carbohydrate chain occurs due to the addition of lactosamines, which can be terminated by structures AB0 and Lewis antigenic determinants, including Le^x^. Interaction with sulfated glycans confirms that the lectin belongs to PRR. It is known that polysaccharides of marine bacteria from shallow-water sediments are often charged with non-carbohydrate substances (sulfates, phosphates, etc.) [52,53,54,55]. We believe that the antibacterial activity of lectin may be mediated by interactions with sulfated glycans on the cell surface of these bacteria.

Thus, the results of the glycan array are in good agreement with the previously obtained data on GYL carbohydrate specificity.

## 3. Materials and Methods

### 3.1. cDNA Sequences Determination and Phylogenetic Analysis

Fresh hemocytes of the bivalve mollusk G. yessoensis samples were used to isolate total RNA by ExtractRNA solution (Evrogen, Moscow, Russia) and applied for the preparation of full-length-enriched cDNA using Mint cDNA synthesis Kit (Evrogen, Moscow, Russia). The rapid amplification of cDNA 3′-ends (3′-RACE) was carried out with the forward primers Lectin_PTSE_For in Step 1, Lectin_MMD in Step 2 (Table 3), and the RACE primer set (Evrogen, Moscow, Russia). The primers were designed based on the known partial amino acid sequences of GYL peptide [27]. 5′-RACE was carried out using the reverse primers GYL_Rev1_Cap, GYL_Rev2_T7, GYL_Rev3_Na21 (Table 1), created based on the obtained 3′-RACE sequences, and the RACE primer set (Evrogen, Moscow, Russia). The cDNA full-length sequences amplification was performed using PCRs with GYL_start and GYL_stop primers (Table 1), designed based on obtained 3′- and 5′-RACE sequences. Encyclo^®^ DNA Polymerase (Evrogen, Moscow, Russia) was used for all conducted PCRs. Synthesis of all primers was performed by Evrogen (Moscow, Russia). PCR fragments were subjected to gel electrophoresis analysis, purification, cloning with InsTAclone PCR Cloning Kit (Thermo Fisher Scientific, Waltham, MA, USA), and transformation into DH5α *E. coli* cells (Thermo Fisher Scientific, Waltham, MA, USA) according to manufacturer protocols. PCR products from positive colonies were sequenced using the SeqStudio Genetic Analyzer (Thermo Fisher Scientific, Waltham, MA, USA) with M13 universal primers.

The GYL cDNA full-length sequences were aligned by MEGA 11 [56] software using the Clustal W algorithm with bivalve mollusks sequence homologs searched for in the GeneBank database (http://www.ncbi.nlm.nih.gov/BLAST, accessed on 7 November 2022). Visualization was performed via the software CLC Main Workbench (Qiagen, Aarhus, Denmark).

### 3.2. Bioinformatics Analysis

The physical and chemical properties of the encoded protein were predicted using the online Expasy server (http://web.expasy.org/protparam/, accessed on 7 November 2022). To search for a signal peptide, SignalP 6.0 was used (http://www.cbs.dtu.dk/services/SignalP/, accessed on 7 November 2022), and the three-dimensional (3D) structure of the protein was predicted using the SWISS-MODEL server (https://swissmodel.expasy.org/, accessed on 22 November 2022). The quality of the constructed theoretical protein model was assessed by QMEANDisCo, Ramachandran plot, and Z-score using SWISS-MODEL Validation online tool (http://swissmodel.expasy.org/, accessed on 22 November 2022). Functional domain was predicted by the Simple Modular Architecture Research Tool (SMART) program (http://smart.embl-heidelberg.de/, accessed on 7 November 2022). The putative characteristics of the secondary structure were predicted by the PredictProtein server (https://www.predictprotein.org/, accessed on 18 November 2022) from the GYL amino acid sequence. Prediction of GYL glycosylation sites from amino acid sequence data was performed using the NetNGlyc-1.0 server (https://services.healthtech.dtu.dk/service.php?NetNGlyc-1.0, accessed on 18 November 2022), which predicts N-glycosylation sites in proteins using artificial neural networks.

### 3.3. N-Terminal Amino Acid Sequencing

SDS-PAGE and the subsequent electrical transfer of GYL onto a polyvinylidene difluoride membrane (Immobilon-P, Merck Millipore, MA, USA) were performed. The amino acid sequence of detected band after staining with Ponceau S solution was determined by Edman degradation using a protein sequencer PPSQ-33A (Shimadzu, Kyoto, Japan). Analysis was performed according to the manufacturer’s protocol. Amino acid residues were identified as phenylthiohydantoin (PTH) derivatives using LabSolutions version 1.01 (Shimadzu, Kyoto, Japan) software.

### 3.4. Sugar Analysis

An aliquot of GYL sample (0.1 mg) was subjected to a methanolysis reaction with HCl/CH3OH (1.25 M, 1 mL) at 80 °C for 16 h. The methanol evaporation acetylation of the methyl glycosides was performed with acetic anhydride in pyridine at ambient temperature for 16 h. The acetylated methyl glycosides were subjected to GS-MS using a Hewlett Packard 5890 chromatograph (USA) equipped with an HP-5MS capillary column and a Hewlett Packard 5973 mass spectrometer (USA). The following temperature program was applied to analyze the derivatives: 150 °C for 3 min, 150 °C → 250 °C at 3 °C/min, and 250 °C for 10 min.

### 3.5. Determination of Fine Carbohydrate Specificity

The fine carbohydrate specificity of the lectin toward mammalian glycans and bacterial polysaccharides was determined using a glycan microarray constructed by Semiotik LLC (Moscow, Russia). Pure GYL was labeled with the biotin according to the product manual. The array was composed of more than 600 tested glycans, each in 6 replicates. The amount of biotinylated GYL bound to each glycan was determined after adding FITC-labeled streptavidin. Slides were scanned using an InnoScan1100 AL (Innopsys, Carbonne, France) equipped with a 488 nm laser at 100 PMT and a high-power laser mode. The data were processed by fixed 100 μm diameter ring method using Mapix 7.3.1 and Mapix 8.2.2 software (Innopsys, France). Data were presented as the relative fluorescence units (RFU) median of six spot replicates. The signals with fluorescence intensity over the background value by a factor of five passed as significative.

## 4. Conclusions

In the present study, a full-length cDNA sequence encoding a novel lectin from the bivalve *Glycymeris yessoensis* was determined for the first time. Analysis of the GYL amino acid sequence revealed that it was a CTLD-containing lectin having various isoforms, which were named GYL-like 1–8. This is the first reported case of the discovery of a multigene family of C-type lectins in bivalves. We determined that the lectin is a glycoprotein with two glycosylation sites per GYL subunit. A study of the GYL glycan-binding activity and structure revealed that it belongs to α/β-structured proteins and exhibits strict specificity toward the constructed epitope “Galβ1-4GlcNAcβ obligatory containing an additional lectin contacting fragment”. It has not yet been established whether the natural ligand of GYL is found within the organism itself or in the surrounding environment. However, the physiological roles of this protein will remain unclear until their target glycans are identified. One possibility is that yet-unknown cellular targets with a surface expression of these sugar chain structures are present in marine environments occupied by mollusks and that GYL recognizes and inhibits the proliferation of such targets.

## Figures and Tables

**Table 1:** Predicted properties and secondary structure analysis of GYL-like proteins.

The GenBank Accession Numbers	Name	Molecular Weight Mature Proteins	pI	Components of the Secondary Structure of GYL-like CTL		
				α-Helix, %	β-Strand, %	Coil, %
OP897819	GYL	16,046.59	4.6	17.02	23.40	59.57
OP897820	GYL-like 1	16,482.77	5.15	15.07	26.71	58.22
OP897821	GYL-like 2	16,424.74	5.32	15.75	26.71	57.53
OP897822	GYL-like 3	16,862.01	4.39	17.01	22.45	60.54
OP897823	GYL-like 4	15,996.31	4.56	17.27	26.62	56.12
OP897824	GYL-like 5	16,095.44	4.65	17.27	25.90	56.83
OP897825	GYL-like 6	16,081.42	4.65	17.27	25.90	56.83
OP897826	GYL-like 7	16,144.37	4.71	17.27	28.78	53.96
OP897827	GYL-like 8	16,042.21	4.65	17.39	27.54	55.07

**Table 2:** GYL binding signals to glycans.

#	Glycan Structure	Short or Trivial Name	Average RFU
404	GalNAcα1-3**Galβ1-4**(Fucα1-3)**GlcNAcβ**-sp3	GalNAcα3′Le^x^-C3	9114
159	4-O-Su-**Galβ1-4GlcNAcβ**-sp3	4′-suLN-C3	5774
183	4,6-O-Su2-**Galβ1-4GlcNAcβ**-sp2	4′,6′-su2LN-C2	3338
368	GalNAcα1-3(Fucα1-2)**Galβ1-4GlcNAcβ**-sp3	A (type 2)-C3	2080
97	**Galβ1-4GlcNAcβ**-sp3	LN-C3	929
234	**Galβ1-4**(Fucα1-3)**GlcNAcβ**-sp3	Le^x^-C3	742
275	GalNAcβ1-3**Galβ1-4GlcNAcβ**-sp3	GalNAcβ3′LN-C3	615
Selective GYL (50 µg/mL) binding entities, including a serial number of each glycan (#), glycan structure, and binding signals in relative fluorescence units (average RFU) in decreasing order.			

**Table 3:** Primers used in this study.

Primers Name	Sequence (5′–3′)	PCR Objective
Lectin_PSTE_For	AGCNCARCCNCANWSNGARGG	3’-RACE
Lectin_MMD_For	ATGATGGAYTAYGTNRAYTGGATG	
GYL_Rev1_Cap	ACATCTTTCCGGTCAATGACTAT	5’ RACE
GYL_Rev2-T7	ACTATATTACCGATCCCAAGGAGT	
GYL1_start	CAGTTTATTGAGGAAGGAGACTTG	PCR of full-length cDNA
GYL1_stop1	CAATGACTATATTACCGATCCCAA	
M13 F	GTAAAACGACGGCCAGT	Sanger sequence of clones
M13 R	CAGGAAACAGCTATGAC	

## Data Availability

Data available on request.
